# Molecular and Antigenic Characterization of GI-13 and GI-16 Avian Infectious Bronchitis Virus Isolated in Chile from 2009 to 2017 Regarding 4/91 Vaccine Introduction

**DOI:** 10.3390/ani9090656

**Published:** 2019-09-05

**Authors:** Miguel Guzmán, Leonardo Sáenz, Héctor Hidalgo

**Affiliations:** 1 Programa de Doctorado en Ciencias Silvoagropecuarias y Veterinarias, Campus Sur Universidad de Chile, Santa Rosa 11315, La Pintana, Santiago 8820808, Chile; 2 Laboratory of Avian Pathology, Faculty of Veterinary and Animal Sciences, Universidad de Chile, Santiago 8820808, Chile; 3 Laboratory of Veterinary Vaccines, Faculty of Veterinary and Animal Sciences, Universidad de Chile, Santiago 8820808, Chile

**Keywords:** 4/91 vaccine, antigenic relationship, infectious bronchitis, phylogenetic classification, protection test, RT-qPCR

## Abstract

**Simple Summary:**

The high adaptation and recombination abilities of infectious bronchitis virus (IB) have been proven. This study aims to verify the genetic and antigenic variation of eight field IB strains regarding the 4/91 strain vaccination in Chilean chickens. Phylogenetic, serologic and challenge studies were carried out to accomplish this goal. The genetic analyses indicate that all the viruses isolated prior to the 4/91 introduction belong to the genetic group GI-16 (three isolates from 2009). On the other hand, just one of the viruses isolated after the 4/91 strain vaccine introduction in Chile (in 2015) showed relationship with GI-16 lineage. The remaining four viruses (from 2017) belong to GI-13, the group where the strain 4/91 has been previously classified. Three viruses were chosen to perform antigenic and protective studies. Antigenically, the high relationship between the 4/91 vaccine with the isolate from 2017 is remarkable and could not be observed with isolates from 2009 and 2015. The 4/91 vaccine also showed better protection against the isolate from 2017 than isolates from 2009 and 2015. These results suggest that the introduction of the 4/91 vaccine in Chile could imply a change in some viruses, showing its ability to interact with field viruses, so it is important to monitor the circulating viruses and include these results in future governmental decisions.

**Abstract:**

The introduction of the 4/91 vaccine against infectious bronchitis in Chile, a lineage not described until that time in the country, led to looking for changes induced by this action. This study considers eight isolates obtained from 2009, 2015 and 2017 and uses a maximum likelihood approach to classify the field isolates. Three isolates were selected to analyze antigenic relationships through a virus neutralization test and to perform protection tests measured trough an RT-qPCR. The isolates from 2009 and 2015 showed a relationship with GI-16 while those from 2017 were related to GI-13. Though the field isolates were classified in two different phylogenetic lineages, all of them showed only minor variations in subtype. The 13885R-17 isolate from 2017 exhibited high antigenic relatedness to the 4/91 vaccine. As expected, 4/91 and Massachusetts vaccines were not antigenically related. Vaccinated birds with the 4/91 vaccine showed less tracheal virus replication for the 13885R-17 from 2017 challenge than for the 12101SP-09 from 2009 and 13347SP-15 from 2015 isolates. The results indicated genetic and antigenic diversity in the most recent infectious bronchitis virus (IBV) isolates in Chile. Moreover, the 4/91 vaccine would be involved in the generation of some current field viruses, which must be considered in vaccination programs and public policies.

## 1. Introduction

A worldwide infectious disease, infectious bronchitis causes huge economic losses in poultry production systems [1]. Its etiological agent is the infectious bronchitis virus (IBV), a Coronaviridae-family gammacoronavirus whose genome is a positive-sense, single-stranded RNA that is 27.6 kb in length [2]. IBV has four structural proteins, membrane (M), nucleocapsid (N), envelope (E) and spike (S). The latter is cleaved into two subunits, where S1—the principal antigen generating neutralizing antibodies—recognizes host cellular receptors and, therefore, determines the virus tropism, and S2 attaches the entire protein to the virus [2]. The non-structural protein 14 of Coronaviruses has a 3′ to 5′ exonuclease activity, which keeps under control the error rates in the replication process, although allowing the generation of diversity for their evolution and adaptation, thus contributing to emergence of new strains [3]. Identification of three hypervariable regions (HVR) along S1 [4,5] is a reasonable way to evaluate structural changes in S1 and thereby determination of the homology between different strains. Understanding the changes in the primary sequence of these regions is an approach to the conformational changes observed in the tertiary one, which finally is responsible for evolution and immune escape of the virus.

IBV control is primarily through vaccines; however, the continuous appearance of new strains in different countries around the world [6,7,8,9,10] necessitates assessment and upgrading of vaccination programs. In some cases, this means widening the protectotype by adding more strains [11]. When IBV was first described in Chile [12], the Massachusetts (Mass) strain was the only vaccine authorized by the Ministry of Agriculture. In 2009, several outbreaks were reported in flocks that had received the Mass vaccine, latter characterized as Q1 strains [13] and classified in G1-16 lineage [14]. Hence, the Ministry of Agriculture approved the introduction of the 4/91 vaccine even though the serotype was not present in Chile.

Several studies have demonstrated the efficacy of protectotype Mass-4/91 against heterologous strains [15,16,17,18,19]. However, the consequences of introducing a vaccinal strain that is not present in the country had been debated until this point. This work aims to determine the antigenic and genetic variability of IBV samples isolated from outbreaks in commercial flocks in 2009, 2015 and 2017 and examine their relationship with the 4/91 vaccine, which was introduced in Chile during that period.

## 2. Materials and Methods 

All embryonated chicken eggs and birds were handled according to national and international animal welfare protocols. All procedures and euthanasia methods were approved by the animal bioethics committee at the College of Veterinary Medicine and Animal Sciences (certification no. 4-2016).

### 2.1. Embryonated Chicken Eggs and Chicks

Embryonated chicken eggs from an experimental SPF flock were used. These hens belong to white leghorn breed, they did not receive any type of vaccine, including IBV, and were kept under strict biosafety conditions. Moreover, the eggs were routinely monitored for IBV antibodies according to the method described by Polson [20]. In brief, the yolk was mixed with chloroform and centrifuged. This procedure was performed twice, then the aqueous supernatant was tested for IBV using a commercial ELISA kit (IDEXX). These eggs were used for virus isolation and incubated to obtain the one-day-old chicks for protection tests and to produce hyperimmune serum.

### 2.2. Viruses

This study involved eight IBVs isolated from different outbreaks in commercial chicken flocks. The isolates 12101SP-09, 12101TR-09, 12124TR-09 were obtained in 2009 when the Mass vaccine was the only live attenuated/inactivated vaccine applied in Chilean poultry. These samples were obtained through tracheal swabs from a 27- to 29-week-old broiler breeder whose flock showed respiratory signs and a drop in egg production.

The 13347SP-15 virus was isolated in 2015, four years after introduction of the 4/91 vaccine in Chilean productive systems. The samples were tracheal swabs from 38-day-old broilers whose flock exhibited inflammation in the infraorbital mucous membrane, bilateral facial edema, abundant palatine and pharyngeal secretion and nephrosis.

Finally, four viruses were isolated in 2017, six years after approval of the 4/91 vaccine. The 13885R-17 and 13861R-17 were isolated from two flock of 35-week-old laying hens exhibiting tracheal secretions and enlarged kidneys, urates in renal parenchyma and uric acid deposits on the pericardium, liver and intestines. The viruses 13691TR-17 and 13698SP-17 were isolated from broiler flocks of 32 and 25 days with tracheal secretions. The samples were tracheal swabs. In addition to the aforementioned samples, the Mass and 4/91 commercial vaccines were also included in the study.

All the sampled flocks belong to the Chilean central zone, where the poultry industry has a major development. The complete zone also had several outbreaks in vaccinated poultry between 2008 and 2010, later described as Q1 strain [13].

Each sample was used to inoculate six 10-day-old embryonated eggs. At 24 h post inoculation (p.i.), non-specific mortalities were removed. Three eggs were sacrificed at 48 h p.i. to obtain chorioallantoic fluid for subsequent passages. Between three and four passages were performed according to Gelb et al. [21] until lesions compatible with IBV—like stunting, curling, clubbing of the down or urate deposits in the mesonephros—were observed [22]. Samples were then confirmed by RT-PCR.

### 2.3. Molecular Characterization

Viral extraction from the samples was performed with a PureLink™ Viral RNA/DNA Mini Kit (Invitrogen, USA) according to the manufacturer’s instructions. The full S1 segment (~1600 bp) was amplified with the IBVS1-F (5′-AACTCTGCACGCAAATTA-3′) and IBVS1-R (5′-TGTTGTCGCAAACAGGACC-3′) primers described by Zhang et al. [23]. 

The RT-PCR was performed using a SuperScript^®^ III One-Step kit (Invitrogen, USA) with the following parameters: 30 min at 55 °C, 2 min at 94 °C, 40 cycles of 15 s at 94 °C, 30 s at 49 °C, finally an extension step at 68 °C for 5 min was added. The RT-PCR products were visualized in an agarose gel then purified using a PureLink™ Quick Gel Extraction Kit according to the manufacturer’s instructions. Finally, an ABI 3730XL genetic analyzer was used in the sequencing process.

The alignment calculation for the nucleotide sequences was performed with Mafft 7.2 software [24] using the L-INS-i method. The nucleotide model substitution was then identified using Jmodeltest software v2.1.7 [25], and a maximum-likelihood tree was constructed with the online platform PhyML 3.0 [26] using the dataset previously published by Valastro et al. [14]. Finally, the amino-acid sequences were aligned using Bioedit v7.2.5 [27].

### 2.4. Virus Neutralization Test

According to the results obtained in the molecular characterization, three isolates were selected considering the lineage and the isolation year. Serotype-specific antisera were prepared in chicks for field viruses 12101SP-09, 13347SP-15 and 13885R-17 as well as the attenuated 4/91 and Mass vaccines purchased from the local MSD representative. The viruses were inactivated with 0.1% v/v formalin at 37% for 12 h. After shaking, they were mixed with complete and incomplete Freund’s adjuvants for the first (at two weeks old) and second (at four weeks old) inoculations, respectively, via subcutaneous. Two weeks after the last inoculation, the chicks were sacrificed and the blood harvested. The sera obtained were inactivated at 56 °C for 30 min, and the antibody levels were evaluated using a commercial ELISA kit (IDEXX).

Cross virus-neutralization tests for Mass, 4/91, 12101SP-09, 13347SP-15 and 13885R-17 were conducted in 10-day-old embryonated eggs. Eight serial ten-fold virus dilutions were prepared with Tryptose Soya Broth (TSB) and mixed in equal proportion with the challenge sera. After 45 min of incubation at 4 °C, five embryonated eggs were inoculated for each dilution. The embryonated eggs were sacrificed seven days p.i. and the lesions recorded. The infectivity titer was calculated to determine the 50% chicken embryo infective dose (EID_50_) according to the Reed–Muench method [28]. Then, values for the neutralizing index (NI) were estimated using the difference between the titers logged for the two strains, relatedness values (R) were calculated using the method of Archetti–Horsfall [29] and classified according to Brooksby criteria [30]. This means, antigenic identity: R~100%; lesser differences: R >70%; minor changes in the subtype: >33% R < 70%; major changes in the subtype: >11% R < 32%; and serotype change: 0% > R < 10%. 

### 2.5. Protection Test

This phase aimed to evaluate the protectivity of 4/91 vaccine by a single administration against the three field isolates under study. Sixty one-day-old SPF chicks were received, and blood samples were drawn and tested for IBV antibodies using an IDEXX commercial kit. Then, all the chicks were immunized with the 4/91 vaccine by nasal instillation with 10^6^ EID_50_/mL.

The chicks were divided equally into four groups, each of which was housed in a different, isolated room with controlled access and high levels of biosafety. At three weeks old, three of the four vaccinated groups were challenged with at least 10^7^ EID50/mL of 12101SP-09, 13347SP-15 or 13885R-17 viruses by nasal instillation. As a non-challenged control group, the last group was inoculated with distillated water.

The health status of the chicks was monitored daily. At six days p.i., they were sacrificed, and tracheal swabs were taken and stored at −80 °C until viral recovery could be quantified through RT-qPCR. Finally, a complete necropsy was performed on all the birds.

### 2.6. Design of RT-qPCR for IBV

Viral extraction was performed with a PureLink™ Viral RNA/DNA Mini Kit (Invitrogen, USA) according to the manufacturer’s instructions. A segment of 72 bp from N gene sequence was amplified using newly developed primers IBVN8-F (5′-CCGAGACCAAAGTCACGCCC-3′) and IBVN8-R (5′-ACGCTGTTGTCTTGGCGCT-3′), and a SensiFast™ SYBR^®^ No-ROX One-Step Kit (Bioline) with the following parameters: 47 °C for 18 min., 95 °C for 5 min and 40 cycles at 95 °C for 10 s, 60 °C for 10 s and 72 °C for 10 s. Standard curves were developed in triplicate with six ten-fold serial dilutions of purified amplicon beginning at 6.6175 × 10^1^ ng/µL through 6.6175 × 10^−6^ ng/µL quantified in an Epoch2 spectrophotometer (BioTek, USA). The efficiency of the reaction was 1.05 while R and R^2^ reached values of 0.99. The melting curve analysis was conducted by raising 0.5 °C between 50 °C and 85 °C, the melt peak was between 82 °C and 82.25 °C and primer-dimers or nonspecific products were not detected.

### 2.7. Statistical Analysis

The Kruskal–Wallis test was used to compare optical density (OD) of the different sera obtained from virus neutralization test. The Shapiro–Wilk test was used to verify normal distribution of cycle threshold (Ct) values of the RT-qPCR from the protection test. Tukey’s multiple comparison test was used to compare Ct values between groups. All the aforementioned tests were performed with RStudio software version 0.98 (RStudio Team, 2015).

### 2.8. Accession Number of Sequence Data

The nucleotides sequence data reported in this paper have been submitted to Genbank database. The accession numbers assigned were KY861928 for 13347SP-09 isolate, KY861929 for 12101TR-09, KY861930 for 12124TR-09, 15KY861931 for the 12101SP-09, MG545597 for 13691TR-17, MG545598 for 13698SP-17, MG545599 for 13861R-17 and MG545600 for 13885R-17.

## 3. Results

### 3.1. Molecular Characterization

**Figure 1:**
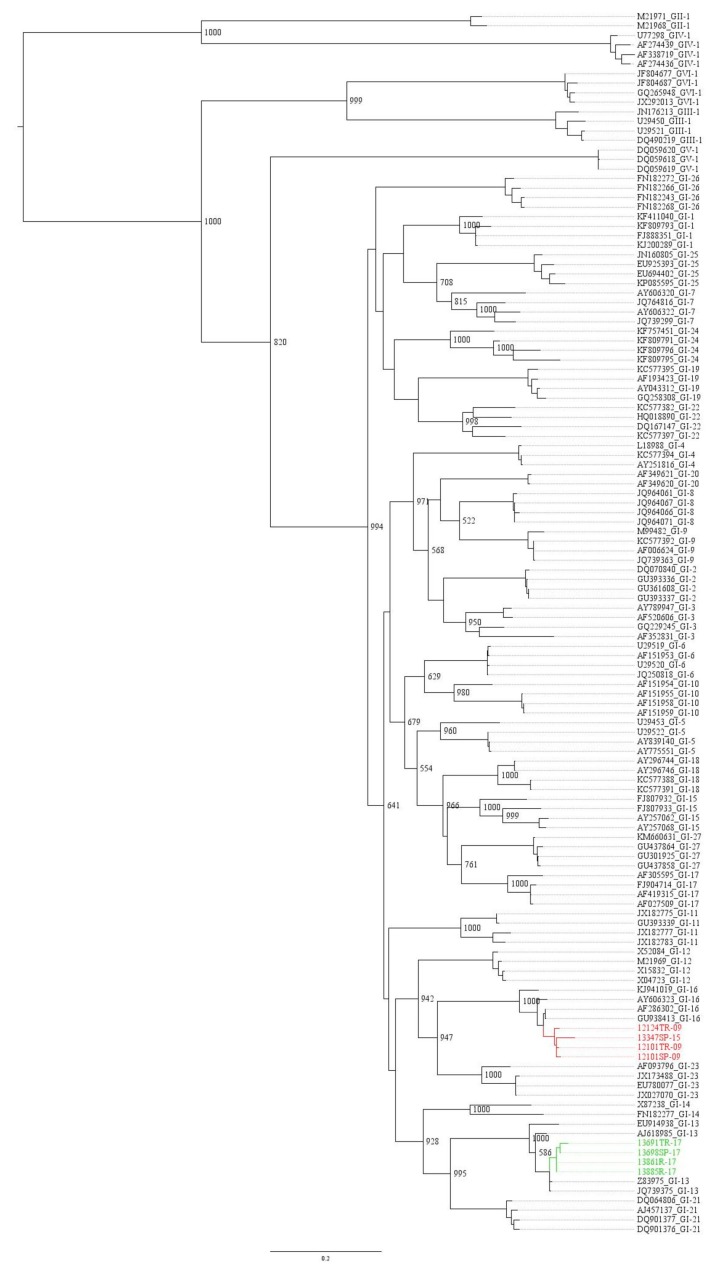
Phylogenetic tree performed by a maximum likelihood method with the PhyML platform v3.0 [26] based on complete S1 region of infectious bronchitis virus (IBV). Red lines indicate Chilean sequences. The tree is rooted in GII and GIV because they showed higher amino-acid distance from other genotypes. The numbers indicate the bootstrap supports of 1000 replicates; only internal nodes over 500 are shown.

The 12101SP-09, 12101TR-09, 12124TR-09 and 13347SP-15 isolates were clustered together in the GI-16 lineage (Figure 1) according to Valastro et al. [14]. These authors included in this lineage strains previously known as Q1, which have been previously described in South America [13,31,32]. However, the isolates 13885R-17, 13861R-17, 13691TR-17 and 13698SP-17 were grouped separately in the GI-13 lineage (Figure 1), with strains described as 793/B, CR/88 or 4/91 [14].

**Figure 2:**
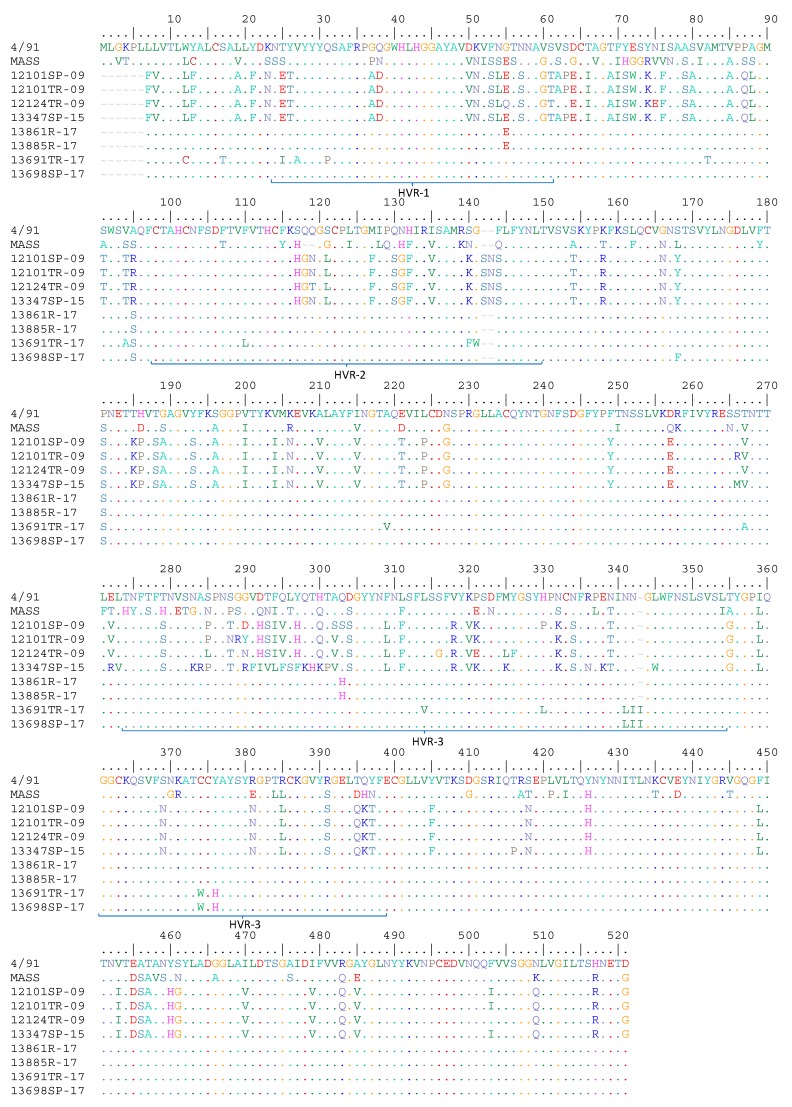
Amino-acid comparison of Mass and 4/91 vaccines with field strains from 2009, 2015 and 2017 along the entire S1 sequence performed with Bioedit software v7.2.5 [27]. Dots indicate the same amino acid in that position compared to the 4/91 vaccine.

Figure 2 shows the amino-acid differences between the field strains and the Mass and 4/91 vaccines currently applied in Chile. Marked differences are observed in the described hypervariable regions in S1 [4,5,33] between strains isolated in 2009 and 2015 with Mass and 4/91 vaccines.

A remarkable similitude between the viruses isolated in 2017 and the 4/91 vaccine is observed, especially with the isolates 13861R-17 and 13885R-17. Only four amino-acid differences—residues 55, 95, 181 and 303 (Figure 2)—could be verified along the whole S1 amino-acid sequence. Similarly, [34] found three amino-acid differences between a vaccine-like virus and the 4/91 vaccine. A change in position 55 (counting from the start of the open reading frame of the S1 gene) from glycine to glutamic acid was identified. 

Though the 13885R-17 and 13861R-17 viruses were isolated from 35-week-old laying hens 21 weeks after the last application of the IBV live attenuated vaccine, and the lack of virulence markers remains as a current issue [35], it is not presumed to be a vaccine re-isolation. Despite de Wit [36] having reviewed field viruses or vaccines recovery up to 28 weeks, this long period is mainly from feces, and the viral recovery from tracheas and kidneys is significantly lower [37,38]. Cavanagh et al. [39] showed that passages in chicks produce a change in position 95, from alanine to serine, as observed here. Furthermore, these isolates were obtained from an outbreak in a commercial flock exhibiting clinical signs, they were not fortuitous isolations. Finally, the flock was negative for most common respiratory pathogens. Thus, the 13885R-17 and 13861R-17 isolates appear to be wild viruses derived of the 4/91 vaccine, emerging after an unquantified number of passages within the flock. 

Because of the results obtained in the phylogenetic study, which clustered into two lineages of the field viruses, and considering different years of isolation, three field viruses were chosen to develop the virus neutralization and protection test. The 12101SP-09 isolated before the 4/91 introduction and the viruses 13347SP-15 and 13885R-17 isolated after the 4/91 introduction [40].

### 3.2. Virus Neutralization Test

ELISA tests detected neutralizing antibodies in all of the chicks inoculated with the field viruses as well as the 4/91 and Mass vaccines. All the samples reached S/P values over the cut-off point provided by the kit manufacturer (Table 1). Thus, it was possible to prove that the production of hyperimmune serum had been successful and that there was not a significant difference in the levels of optical density achieved (*p*-value = 0.25) in the different groups.

Brooksby’s criterion [30] was used to classify the antigenic relationships, unveiled with the Archetti-Horsfall method [29], between the field strains and the Mass and 4/91 vaccines. Minor changes in the subtype were detected between virus 12101SP-09 and the Mass and 4/91 vaccines (Table 2). The outbreaks between 2008 and 2010 occurred in flocks that had previously been vaccinated with the Mass strain. Nonetheless, previous assays had shown that the efficiency of the Mass-4/91 protectotype justified introduction of this scarcely related vaccination. The antigenic relationship observed between the 12101SP-09 strain and the two vaccines was also low. Finally, among the field strains, minor changes in subtype were shown for all the reactions.

On the other hand, the high antigenic relationship observed between virus 13885R-17 and the 4/91 vaccination is noteworthy. Using Brooksby’s criterion [30], only minor changes were found between the two. These results align with those presented by Moore et al. [41], which showed that at least the amino acid 304 residue would be related to the neutralizing serotype-specific epitope. In this study, the 4/91 vaccine and 13885R-17 virus showed a high antigenic relationship and were also shown to share an aspartic acid in position 304—as well the other viruses isolated in 2017—rather than the serine found in the other field viruses (Figure 2). As such, the results presented here support the findings published by Moore et al. [41], where the authors show that changes in just one or two specific residues are enough to neutralize or not neutralize a specific strain.

### 3.3. Protection Test

An ELISA test performed on the one-day-old chicks used for the protection tests showed that they did not have IB-neutralizing antibodies prior to vaccination. The control group did not show clinical signs, and the presence of the virus was not detected through RT-qPCR. All of the birds challenged with the 12101SP-09 and 13347SP-15 viruses showed clinical signs compatible with IB at 48 h p.i., mainly inflamed infraorbital sinuses and tracheal rales. In addition, during the necropsy the birds challenged with strain 13885R-17 showed lesser presence of mucosity in the upper respiratory tract compared to that observed with the other two groups, which presented a large amount of mucosity. The remaining organs did not exhibit any apparent lesion. In addition, those challenged with 13885R-17 did not show any clinical signs during the entire assay. Results are summarized in Table 3.

Table 4 presents the Ct values of the RT-qPCR performed from tracheal swabs from birds challenged with the three field viruses. The values ranged between 25 and 33.45 for the group challenged with the strain 12101SP-09, meanwhile between 26.32 and 32.13 from the chickens challenged with the virus 13347SP-15, finally, a higher range was registered from the group challenged with the virus 13885R-17, between 32.02 and 36.59 Ct values. These values are in accordance with the results presented by Fellahi et al. [42] in a validation for an RT-qPCR targeting 130 bp of N gene.

**Figure 3:**
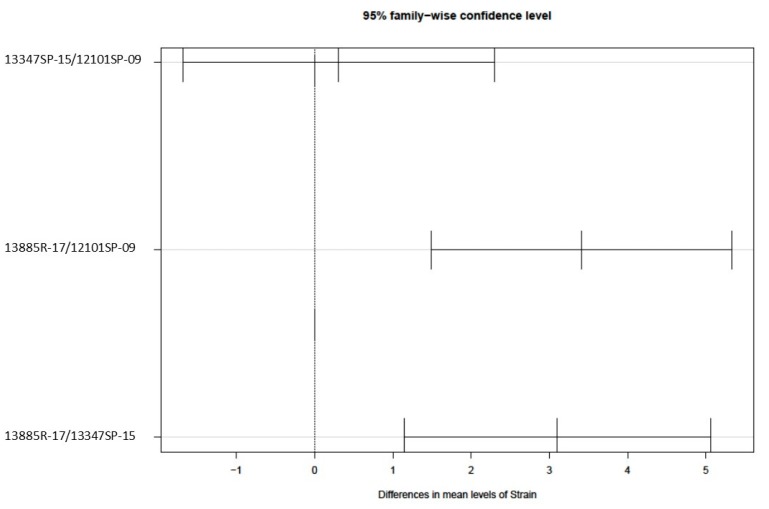
Results of Tukey’s multiple comparison test (*p*-value = 0.05). Intervals that do not overlap the dotted line indicate a significant difference between the Ct values of the RT-qPCR from different challenge strains.

The Ct values showed a normal distribution (Shapiro–Wilk test *p*-values of 0.5624). There was no difference in the Ct values of the RT-qPCR performed from the groups of birds challenged with strains 12101SP-09 and 13347SP-15 (*p*-value = 0.9252) (Figure 3). On the other hand, the Ct values of the RT-qPCR from the swabs obtained from birds challenged with virus 13885R-17 were significantly higher than the amount recovered from groups challenged with the 12101SP-09 (*p*-value = 0.000299038) and 13347SP-15 (*p*-value = 0.0011961) isolates (Figure 3). This data are in alignment with the clinical signs and lesions observed. Thus, the higher Ct of the RT-qPCR performed from birds challenged with the virus 13885R-17 shows that the samples had a lower initial viral amount, which is because of a more efficient inhibition of viral replication by the 4/91 vaccine.

## 4. Discussion

This study evaluated eight isolates of the infectious bronchitis virus obtained in different years. The analysis of genetic sequences allowed to choose three of them to evaluate antigenic and protectivity changes in relation with 4/91 vaccine. Antigenically, minor variations in subtype were established between the 12101SP-09 isolate from 2009 and the Mass and 4/91 vaccine viruses. The same type of antigenic relationship was described between the 13347SP-15 isolate and both vaccines (Table 2). Field viruses 12101SP-09 and 13347SP-15 belong to the same GI-16 lineage (Figure 1). As such, they are expected to share the same type of antigenic relationship with the vaccines despite being isolated six years apart, thus, the 23 different residues observed (Figure 2) were not enough to cluster apart the viruses from 2009 and 2015. Thus, we can conclude that the amino-acid changes observed between the two field strains (Figure 2) were not in residues critical to changing their affinity for the Mass and 4/91 vaccines.

The Q1 strains isolated from China have shown mainly respiratory signs and diarrhea plus proventriculus lesions [43]. In the other hand, Chilean Q1 strains exhibited respiratory signs and renal lesions, although the virus could not be isolated from proventriculus [13]. It was shown that, despite the difference in the biological behavior of the Chilean viruses and the Q1 strains isolated from China, the combination of the Mass and 4/91 vaccines provided good levels of protection against the GI-13 viruses isolated in Chile, supporting the introduction of the 4/91 vaccine in Chile [13]. More recently, de Wit et al. [44] have shown that better levels of protection can be achieved by applying an inactivated IB vaccine after prior stimulation with attenuated Mass and 4/91 vaccines. Along these lines, Chilean poultry producers have adopted the strategy of vaccinating chicks with attenuated Mass and 4/91 vaccines during the rearing period. At the end of that period, they use an inactive Mass vaccine, which has been shown to be effective, given that the clinical outbreaks have mainly been controlled.

However, six years after the introduction of the 4/91 vaccine, viruses isolated in 2017 were classified along with that vaccine in GI-13 (Figure 1). Moreover, the replication of the 13885R-17 virus was more inhibited by the 4/91 vaccine compared to the viral replication obtained with the 12101SP-09 and 13347SP-15 viruses (Figure 3). The results presented here are consistent with those of Liu et al. [45], who showed that birds vaccinated against IB and then challenged with homologous strains were well protected. On the other hand, if the challenge was with a heterologous strain, protection was generally variable and lower.

As expected, it was possible to verify that the recently introduced 4/91 vaccine provides better protection against the strain isolated in 2017, to which it is more genetically and antigenically related. Immune response would be mainly responsible for this different protection level, however, it will be measured to elucidate the specific role of the avian immune system [46,47,48]. Although there is no discussion of whether the introduction of the 4/91 vaccine was appropriate for controlling the clinical problems observed in the field, the appearance of the GI-13 lineage re-opens the question regarding the anthropomorphic role in disseminating different strains in locations where they have not been described naturally.

Cook et al. [16] proposed a new strategy for addressing the constant emergence of new strains—using two heterologous vaccines to develop protectotypes in order to stimulate immune response and neutralize different pathogenic strains. While the first combination described was using Mass and 4/91 vaccine strains, other combinations could also be effective. As such, studying the protectiveness of the vaccines through the concept of protectotype rather than evaluating serotypes or genotypes presents as a reasonable solution. However, constant, objective elucidation of the relationship between genotype, serotype and protectotype is vital as, in some cases, minimal changes in one of these characteristics can produce significant changes in another [49].

The increase in protectiveness granted through the combination of the heterologous Mass and 4/91 vaccines has already been proven [15,16]. As a result, the 4/91 vaccine has been introduced in different countries even when the pathogen variant has not been previously described there [14]. Furthermore, the time and resources required for vaccine development make continuous generation of new vaccines seem inviable at the commercial scale [45].

To date, the IBV vaccine attenuation process involved passage through embryonated eggs, and little is known about the amino-acid residues involved in the loss of virulence. Shimazaki et al. [34] found the same aminoacidic change in the position 55 in a similar field isolate, thus, it could be involved in the virulence reversion of the vaccine. Oade et al. [50] found that the embryonated egg attenuation process can generate different genomic patterns in different attenuated viral populations and verified a low level of consensus among them. As such, the reversal of virulence can be achieved in different and unpredictable ways facilitated by the high rates of mutation described for IBV [31,51,52]. These data support growing concern about introduction of an attenuated vaccine where its pathogenic variant has not been previously described.

## 5. Conclusions

This study is the initial approach to understanding whether the recently introduced 4/91 vaccine has the capacity to modify at least part of the viruses circulating in the field. The local sanitary services should consider the information here showed before authorizing the introduction of foreign strains in their countries. However, the previous statement implies to develop new approaches for vaccines elaboration instead the use of current vaccines, reminding to keep our poultries health now, but without forgetting that it must be in a long-term way too. For more comprehensive understanding, a whole-genome approach must be taken to clarify whether the proximity to the field strains from 2017 is due to vaccine reversion by mutation and/or one or more recombination processes is involved, where the vaccinal strain has been proven to have escaped and contributed with genetic material to field viruses [32,53,54]. A deep epidemiological approach will also be required to evaluate the current status of viral populations so as to determine how many strains are co-circulating and any possible dominance hierarchies established among them.

## Figures and Tables

**Table 1:** Chick antibody levels against different IBV isolated in different years, inoculated at 14 and 28 days old. Optical density (OD) and sample to positive ratio (S/P) values are presented.

Strain	OD Values	S/P Ratios ^a^
**Negative control**	**0.044**	
**Negative control**	**0.046**	
**Positive control**	**0.225**	
**Positive control**	**0.227**	
Mass	0.192	0.875
	0.125	0.476
	0.132	0.518
	0.155	0.655
	0.137	0.548
4/91	0.100	0.306
	0.103	0.324
	0.106	0.335
	0.89	0.243
	0.85	0.220
12101SP-09	0.148	0.598
	0.211	0.982
	0.197	0.880
	0.237	1.14
	0.256	1.256
13347SP-15	0.194	0.864
	0.221	1.023
	0.192	0.875
	0.236	1.112
	0.205	0.929
13885R-17	0.144	0.573
	0.148	0.598
	0.208	0.963
	0.220	1.037
	0.288	1.451

^a^ The ELISA sample to positive (S/P ratio). Samples > 0.2 are considered positive according to IDEXX guidelines. The blood samples were collected two weeks after the second inoculation.

**Table 2:** Archetti and Horsfall antigenic relatedness (R) calculated on embryonated eggs.

Virus Strain	Antiserum				
	Mass	4/91	12101SP-09	13347SP-15	13885R-17
Mass	100	32	41	42	38
4/91	-	100	34	33	73
12101SP-09	-	-	100	53	62
13347SP-15	-	-	-	100	57
13885R-17	-	-	-	-	100

**Table 3:** Clinical signs and lesions at necropsy recorded six days post inoculation (p.i.) in chicks vaccinated with 4/91 and challenged with three different IBV isolates from different years.

Virus Challenged	Clinical Signs and Lesions at Necropsy ^a^			
	Inflamed Infraorbital Sinuses	Tracheal Rales	Tracheal Exudate	Palatine Exudate
12101SP-09	++	+++	+++	+++
13347SP-15	++	+++	+++	+++
13885R-17	-	-	+	+

**^a^** The symbology regarding to signs/lesions means: “+++”: High presence; “++”: Medium presence; “+”: Low presence; “-“: Absence.

**Table 4:** Average Ct values of RT-qPCR from tracheal swabs of chicks challenged with three different isolates from different years.

Strain	Ct
12101SP-09	32.53
	25
	28.68
	29.19
	33.45
	31.96
	30.4
	31.85
	35.61
	31.01
	26.47
	29.83
	29.77
	27.18
	32.53
13347SP-15	29.63
	30.11
	30.06
	30.85
	31.66
	26.32
	31.77
	31.19
	34.43
	32.13
	29.92
	28.26
	30.39
	29.63
13885R-17	36.59
	34.34
	32.07
	32.36
	32.67
	32.77
	33.48
	3467
	32.58
	33.82
	34.58
	34.99
	32.02
	34.73
